# Functional and topological analysis of PSENEN, the fourth subunit of the γ-secretase complex

**DOI:** 10.1016/j.jbc.2023.105533

**Published:** 2023-12-10

**Authors:** Lutgarde Serneels, Leen Bammens, An Zwijsen, Alexandra Tolia, Lucía Chávez-Gutiérrez, Bart De Strooper

**Affiliations:** 1 Laboratory for the Research of Neurodegenerative Diseases, VIB Center for Brain & Disease Research, VIB, Leuven, Belgium; 2 Department of Neurosciences and Leuven Brain Institute, KU Leuven, Leuven, Belgium; 3 Laboratory of Developmental Signaling, Center for Human Genetics, KU Leuven, Leuven, Belgium

**Keywords:** PSENEN, presenilin, γ-secretase, Alzheimer

## Abstract

The γ-secretase complexes are intramembrane cleaving proteases involved in the generation of the Aβ peptides in Alzheimer’s disease. The complex consists of four subunits, with Presenilin harboring the catalytic site. Here, we study the role of the smallest subunit, PSENEN or Presenilin enhancer 2, encoded by the gene *Psenen*, *in vivo* and *in vitro*. We find a profound Notch deficiency phenotype in *Psenen*^*−/−*^ embryos confirming the essential role of PSENEN in the γ-secretase complex. We used *Psenen*^*−/−*^ fibroblasts to explore the structure–function of PSENEN by the scanning cysteine accessibility method. Glycine 22 and proline 27, which border the membrane domains 1 and 2 of PSENEN, are involved in complex formation and stabilization of γ-secretase. The hairpin structured hydrophobic membrane domains 1 and 2 are exposed to a water-containing cavity in the complex, while transmembrane domain 3 is not water exposed. We finally demonstrate the essential role of PSENEN for the cleavage activity of the complex. PSENEN is more than a structural component of the γ-secretase complex and might contribute to the catalytic mechanism of the enzyme.

γ-Secretase is a membrane-embedded aspartic protease composed of four subunits: Presenilin 1 or 2 (PSEN1 or PSEN2), Nicastrin (NCSTN), APH1A or APH1B, and PSENEN (1). γ-Secretase is an attractive drug target for Alzheimer’s disease because it is responsible for the final cleavage in the processing of the amyloid precursor protein (APP) to Aβ peptides (2). However, owing to its broad substrate specificity, general inhibition of γ-secretase is not without risks. The main problem is the involvement of γ-secretase in the cleavage of Notch (3, 4, 5), which releases the Notch intracellular domain (NICD) that is implicated in crucial signaling processes throughout life. The essential role of γ-secretase in early development is illustrated by the embryonic lethality of *Drosophila melanogaster*, *Caenorhabditis elegans*, or *Mus musculus* in which *Ncstn*, *Aph1A,* or the two *Psens* together are genetically inactivated. In all cases a severe Notch deficiency phenotype is observed (5, 6, 7, 8, 9, 10, 11, 12, 13, 14, 15). However, it should be noted that other single knockouts of subunits of the γ-secretase complex, like *Psen2* alone (7, 10) or *Aph1B* (8, 16), do not result in Notch phenotypes. It is also increasingly clear that γ-secretases play a role in various other signaling pathways (17). The effect of a genetic knockout of *PSENEN* causing a Notch deficient phenotype is reported here, confirming our retracted report (18).

The elucidation of the structure of γ-secretase (19) has provided a quantum leap in our understanding of these enzymes. The tetrameric γ-secretase encompasses 20 transmembrane domain helices, organized in a horseshoe conformation (20). The catalytic aspartates D257 and D385 are located on transmembrane domain TM 6 and TM7 (21) of the PSEN subunit. The large ectodomain of NCSTN overlays the whole hydrophobic domain of γ-secretase at the extracellular surface. The catalytic aspartates are present in a water-containing cavity to which other parts of PSEN contribute as well (22, 23, 24). The role of the other three subunits of γ-secretase and their contribution to the catalytic activity of the protease are less well understood, although NCSTN is involved in substrate binding (25). All subunits are needed to generate a mature tetrameric γ-secretase complex. In the current work we focus on the smallest subunit, presenilin enhancer 2 or PSENEN, previously called PEN-2.

*Psenen* was originally identified in a genetic screen for modulators of PSEN activity in *C. elegans* (11). PSENEN is a 101-amino-acid-long protein with three hydrophobic domains. Initially several publications suggested a hairpin topology for the protein, with the loop domain exposed to the intracellular side of the cell membrane (18, 26, 27). The cryo-EM structure of γ-secretase (20) has shown, however, that the two first hydrophobic domains of PSENEN make a reentry hairpin loop, resulting in the exposure of its N terminus to the cytoplasmic side of the membrane and the C terminus to the luminal or extracellular side of the membrane. This was also confirmed by other approaches (28).

RNAi-mediated downregulation of PSENEN levels in cell culture leads to decreased endoproteolysis of PSEN, which is associated with an increase of full-length PSEN and a decrease of PSEN amino- and carboxy-terminal fragments (PSEN NTF and PSEN CTF) (29, 30, 31, 32). Additionally, mutational analysis has shown that the N-terminal part of hydrophobic domain 1 of PSENEN interacts with the TMD4 of PSEN1 and is important for PSEN endoproteolysis (31, 33). These observations suggest that PSENEN is involved in the endoproteolysis of PSEN and therefore in the activation of the γ-secretase complex (34). Furthermore, a conserved amino acid sequence motif, DYSLF, in the carboxy terminus of PSENEN, as well as the length of this part of the protein, appears crucial for the assembly of the γ-secretase complex, the stabilization of the PSEN fragments after endoproteolysis, and γ-secretase activity (31, 35, 36, 37). Incorporation of a Flag-tag at the N terminus of PSENEN changes the conformation of PSEN, resulting in an increased Aβ_42_/Aβ_40_ ratio (38), similar to what is observed for familial Alzheimer’s disease mutations in the PSEN subunit (15). Furthermore, a γ-secretase modulator that decreases Aβ_42_ production binds mainly to PSENEN (39), further arguing for the crucial role of PSENEN in the regulation of the activity of the complex.

We confirm the conclusions from our previous report (18) that PSENEN is essential for the γ-secretase processing of Notch and APP. In the absence of PSENEN, no processing of APP is observed, which contrasts with *Ncstn*^*−/−*^ cells, which retain 5 to 6% of γ-secretase activity (40). We made use of the *Psenen*^*−/−*^ fibroblasts to perform a cysteine scanning analysis (41) of the PSENEN protein (22, 23, 24) to investigate its topology and the exposure of individual amino acids to water.

## Results and discussion

### Psenen^−/−^ embryos display a Notch deficiency phenotype

We generated a *Psenen*^*−/−*^ mouse model by crossing *Psenen*^*+/−*^ mice obtained from the Texas Institute for Genomic Medicine. The *Psenen* gene was inactivated by exchanging three of the four *Psenen* exons (exon 2, 3, and 4) for a *LacZ/Neomycin* cassette. *Psenen* gene disruption resulted in embryonic lethality, confirming the essential role of PSENEN in the maturation and/or stabilization of the γ-secretase complex. Up to E9.5, living *Psenen*^−/−^ embryos were recovered in a nearly Mendelian ratio (21/93), while they were completely absorbed by E11.

**Figure 1:**
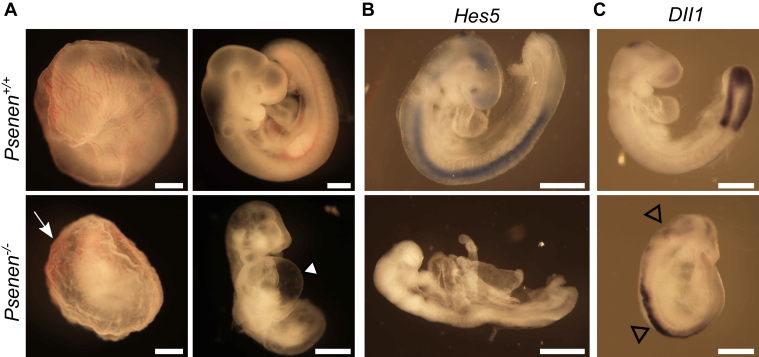
**Notch deficiency phenotype in *Psenen***^***−/−***^**embryos.***A*, the yolk sac (E9.5) of a *Psenen*^*+/+*^ has a smooth appearance with a hierarchically organized vascular network (*upper left panel*). In *Psenen*^*−/−*^ (*lower panel*), an initial vascular plexus and primitive red blood cells have formed (*arrow*) in the yolk sac, but organization into a discrete network of vitelline vessels is lacking. *Psenen*^*−/−*^ embryos (E9) are developmentally delayed. The knockout embryos are smaller than wildtype littermates and characterized by a posterior truncation, a large pericardial sac (*solid arrowhead*), and a distorted neural tube. *B* and *C*, mRNA expression of two target genes of Notch, *Hes5* and *Dll1,* detected by whole mount *in situ* hybridization with digitonin-labeled riboprobes (*blue-brownish color*) shows disturbed Notch signaling in *Psenen*^*−/−*^ embryos. The *Hes5* probe (*B*) reveals repressed *Hes5* expression in *Psenen*^*−/−*^ embryos (E8.5) compared with *Psenen*^*+/+*^ embryos (E8.5) going from the head to the tail along the spinal cord. *Dll1* (*C*) is ectopically expressed in the neural tube and the head in *Psenen*^*−/−*^ embryos (*open arrowheads*). Pictures were taken at different magnifications, represented by scale bars that all represent the same length (approximately 500 μm).

At E9.5, the yolk sac surrounding the *Psenen*^*−/−*^ embryos had only a primitive immature blood vessel-plexus and a blistered appearance compared with the yolk sac of the *Psenen*^*+/+*^ or *Psenen*^*+/−*^ littermates, which displayed a normal complex vasculature (Fig. 1*A*). The *Psenen*^−/−^ embryos were smaller than the *Psenen*^*+/+*^ littermates. They had an abnormally large pericardial sac, a kinked neural tube, and a truncated posterior region. Somitogenesis was initiated in the *Psenen*^*−/−*^ embryos but appeared severely delayed compared with the *Psenen*^*+/+*^ littermates. The optic and otic vesicles, the first branchial arch, and the forelimb buds were visible, but the fusion of the headfolds was delayed (Fig. 1*A*).

The phenotype of the *Psenen*^*−/−*^ embryos was similar to the previously characterized phenotype of the double knockout (dKO) *Psen1&2*^*dKO*^ embryos and is compatible with loss of function of γ-secretase activity and the consequent effects on the Notch signaling pathways (7, 10). To evaluate disturbance in Notch signaling, we performed whole mount *in situ* hybridization on embryos at E8.5 using *Hes-5* and *Delta-like1* probes to detect expression of the respective genes*.* If Notch cleavage by γ-secretase is blocked, then *Hes-5* signals are expected to be downregulated and *Delta-like1* signals to be upregulated, respectively. We observed indeed the absence of *Hes-5* mRNA and ectopic expression of *Delta-like-1* mRNA in *Psenen*^*−/−*^ compared with the *Psenen*^*+/+*^ littermate embryos (Fig. 1, *B* and *C*).

The data confirm that PSENEN is an essential component of γ-secretase. Owing to the severe Notch loss-of-function phenotype, it is difficult to detect potential non-Notch signaling dependent phenotypes. This is like animals with the other γ-secretase components inactivated (7, 8, 9, 10, 16). This precluded assessing the contribution of other γ-secretase substrates in the *Psenen*^*−/−*^ phenotype and to rule out the possibility that PSENEN has additional non-γ-secretase-related functions. However, considering that PSENEN protein expression is strongly dependent on the presence of the three other γ-secretase components (8, 16, 32, 42), it seems logical to assume that PSENEN mainly functions as part of the γ-secretase complex.

### Effect of PSENEN deficiency on γ-secretase activity and assembly

**Figure 2:**
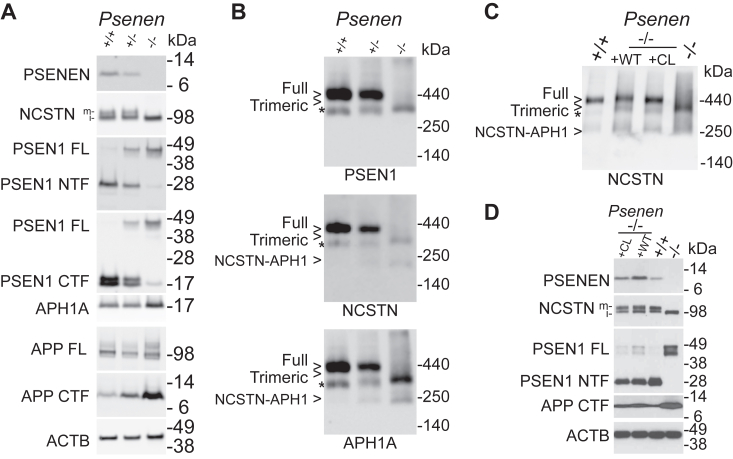
**Characterization of *Psenen***^***+/−***^**and *Psenen***^***−/−***^**fibroblasts.***A*, the 4 to 12% BisTris SDS-PAGE/Western blot analysis of *Psenen*^*+/+*^, *Psenen*^*+/−*^, and *Psenen*^*−/−*^ fibroblasts derived from E9.5 mice. Solubilized membrane protein, 20 μg, was applied per lane, and two gels were run and transferred to nitrocellulose membranes. Based on molecular weight markers the membranes were cut and stained with the indicated antibodies. Membrane 1 was stained with antibody B128 to show PSENEN, with antibody 9C3 to demonstrate immature (i) and mature (m) NCSTN, and Mab5232 to reveal full-length Presenilin 1 (PSEN FL) and C-terminal fragments (PSEN1 CTF). Membrane 2 was stained with B19 to reveal PSEN1 FL and Presenilin1 N-terminal fragments (PSEN1 NTF), B63 was used to stain full-length APP (APP FL) and APP C-terminal fragments (APP CTF), and B80 was used for APH1A. ACTB was used as loading control (result of membrane 2 is presented here). *B*, blue native PAGE/Western blot analysis of Psenen+/+, Psenen+/-, and Psenen−/− dodecylmaltoside extracted membranes (5 μg/lane) stained with antibodies against PSEN1 CTF, NCSTN, and APH1A as in A. Full γ-secretase complex is absent in *Psenen*^*−/−*^ fibroblasts. Two gels were loaded, membrane 1 was first stained with Mab5232 against PSEN1 CTF (*upper panel*) and reprobed with B80 against APH1A (*lower panel*) while membrane 2 was stained with 9C3 against NCSTN (*middle panel*). The trimeric complex in *Psenen*^*−/−*^ fibroblasts is composed of FL PSEN1, NCSTN, and APH1A. ∗ indicates a complex induced by detergent-dependent dissociation, composed of PSEN1 CTF, APH1A, and NCSTN (see ref (66)), which moves slightly faster than the trimeric complex in *Psenen*^*−/−*^. *C*, blue native PAGE/Western blot analysis (5 μg/lane) of membrane fractions of *Psenen*^*+/+*^ fibroblasts*,* of *Psenen*^*−/−*^ fibroblasts reconstituted with wildtype PSENEN (WT) or cysteine-less PSENEN (CL), and *Psenen*^*−/−*^ fibroblasts. The cysteine-less PSENEN mutant is incorporated in the mature γ-secretase complex to the same extent as the wildtype PSENEN. ∗: complex induced by detergent (see *A*). *D*, SDS-PAGE/Western blot analysis of membranes of fibroblasts as in *C* and using the same antibodies as in A. The cysteine-less PSENEN mutant rescues NCSTN maturation and PSEN1 endoproteolysis and restores the cleavage of APP CTF to the same extent as wildtype PSENEN. Samples were loaded on two gels. The first membrane was used to detect PSENEN and ACTB. The second membrane was stained for NCSTN, PSEN1 FL (full length), and NTF (amino-terminal fragment). Afterward this blot was probed for APP CTF.

We derived *Psenen*^*+/+*^, *Psenen*^+/−^, and *Psenen*^*−/−*^ fibroblasts from mouse embryos at E9.5 and immortalized the cells. Western blot analysis of *Psenen*^*−/−*^ cell membrane extracts demonstrated the complete absence of PSENEN protein. In contrast, the three other components of the γ-secretase complex were present at normal levels (*i.e.*, equal to the expression in *Psenen*^*+/+*^ cells) (Fig. 2*A*). Interestingly, PSEN1 was mainly present as full-length protein and NCSTN failed to become fully glycosylated. We observed an accumulation of APP carboxyterminal fragments (CTFs), indicating the γ-secretase activity deficiency in the *Psenen*^*−/−*^ fibroblasts (Fig. 2*A*). γ-Secretase maturation and activity correlated with the expression levels of PSENEN in these cells as assessed in *Psenen*^*+/−*^ cells, suggesting a limiting role of PSENEN in the assembly and activity of the complex in this cell type (Fig. 2, *A* and *B*).

**Figure 3:**
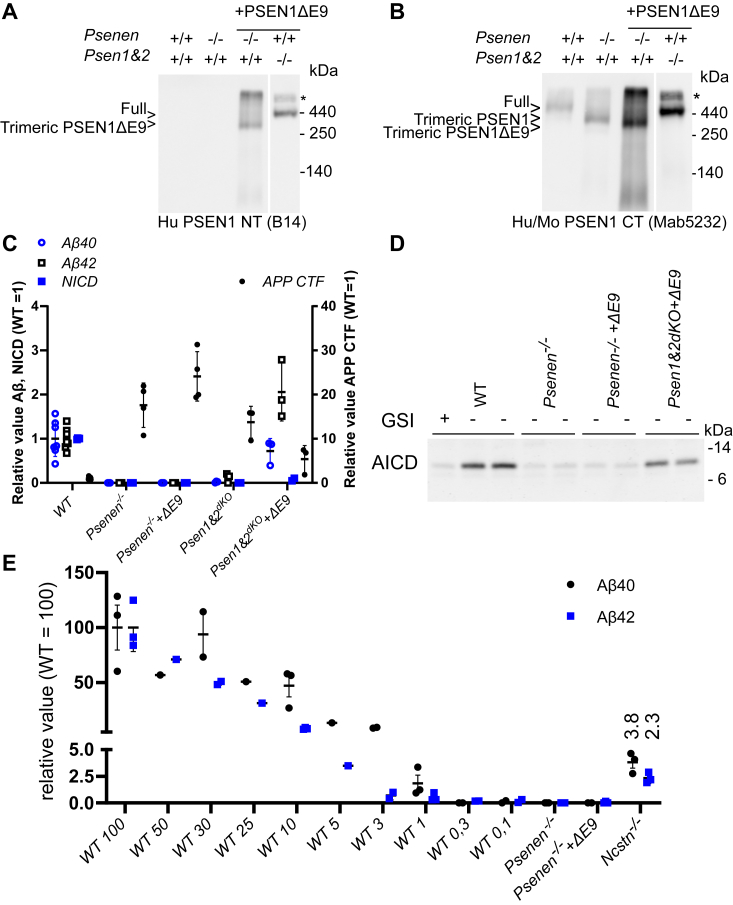
**The lack of γ-secretase activity in *Psenen***^***−/−***^**fibroblasts is not simply caused by a lack of PSEN endoproteolysis.***A* and *B*, blue native PAGE/Western blot analysis of γ-secretase complexes. Twelve micrograms of dodecylmaltoside dissolved membranes from fibroblasts containing WT (+/+) or knockout (−/−) alleles of *Psenen* or *Psen1&2* as indicated. *Psenen*^*−/−*^ fibroblasts and *Psen1&Psen2*^*dKO*^ fibroblasts were transduced with the noncleaved human PSEN1 mutant delta exon 9 (ΔE9). Panel *A* shows the blot stained with human-specific PSEN1 CT antibody (B14) and *B* shows the same blot reprobed with Mab5232, a mouse and human-specific antibody. This shows that PSEN1 ΔE9 is incorporated into the γ-secretase complexes (indicated with full) in the reconstituted *Psen1&2*^*dKO*^ and in the trimeric complex in the absence of PSENEN. ∗ Indicates a nonspecific signal at the upper end of the gel. *C*, *Psenen*^*−/−*^ and *Psen1&2*^*dKO*^ fibroblasts were transduced with PSEN1 ΔE9 as indicated in the figure on the *x*-axis. The γ-secretase substrates APP containing the Swedish mutation or NotchDE were transduced into the fibroblasts, and Aβ_40_, Aβ_42_, and APP CTF or NICD production was assessed by ELISA (Aβ_40,_*blue open circle* and Aβ_42,_*black open square*) or by Western blot (APPCTF, *black circle* and NICD, *blue square*) as indicated in the legend of the graph. None of these γ-secretase products are generated in the *Psen*1&2^dKO^ cells or the *Psenen*^*−/−*^ cells, and an accumulation of APP CTFs is observed. Processing of APP and Notch is partially rescued upon PSEN1 ΔE9 expression in *Psen1&2*^*dKO*^ but not in *Psenen*^*−/−*^. *D*, AICD (the intracellular domain of APP generated by γ-secretase) is generated in microsomal fractions from wildtype fibroblasts (WT) in the presence of APP C99 substrate and is blocked by the addition of 10 μM γ-secretase inhibitor L-685,458 (+GSI). The signal present in lane 1 indicates the background level of the assay. AICD was absent in cell-free reactions using *Psenen*^*−/−*^ microsomal fractions. Expression of human PSEN1 ΔE9 in *Psen1&2*^*dKO*^ restored AICD production, while this is not observed in the *Psenen*^*−/−*^ assay. *E*, ELISA of Aβ_40_ (*black circle*) and Aβ_42_ (*blue square*) of conditioned medium of WT fibroblasts (WT) shows detectable levels of Aβ_40_ signal at 1% of the input material. Thus, Aβ levels in *Psenen*^*−/−*^ fibroblasts are lower than 1% of the Aβ levels in WT fibroblasts. Aβ levels were not detectable in *Psenen*^*−/−*^ fibroblasts rescued with the PS1ΔE9 mutant. Low amounts of Aβ levels in *Ncstn*^*−/−*^ fibroblasts could be detected, in line with previous findings (40) and confirming the sensitivity of the assay.

In blue native PAGE analysis, a trimeric complex was detected in *Psenen*^*−/−*^ fibroblasts that is composed of full-length PSEN1, NCSTN, and APH1A (Fig. 2*B*). Since this trimeric complex contains the catalytic subunit of γ-secretase (PSEN), we wondered whether this subcomplex had some remaining enzymatic activity. We measured Aβ levels in the conditioned medium of *Psenen*^*+/+*^ and *Psenen*^*−/−*^ fibroblasts using the AlphaLISA technology (Fig. 3, *C* and *E*) showing that Aβ_40_ and Aβ_42_ generation was abolished. We also tested the activity of the complex in a cell-free assay by solubilizing microsomal membrane fractions of the *Psenen*^*+/+*^ and *Psenen*^*−/−*^ fibroblasts in 1% CHAPSO and adding recombinant APP-3xFlag substrate (Fig. 3*D*). AICD (the intracellular domain of APP generated by γ-secretase) levels in the *in vitro* reactions were analyzed by SDS-PAGE and Western blotting. This experiment showed that AICD production was reduced to background levels (Fig. 3*D*). Western blot analysis showed that in the *Psenen*^*−/−*^ fibroblasts APP CTFs accumulated (Figs. 2, *A* and *D* and 3*C*). Finally, a dilution series of conditioned medium of *Psenen*^*+/+*^ fibroblasts demonstrated that Aβ_40_ production in *Psenen*^*−/−*^ fibroblasts was less than 1% of the Aβ_40_ production in *Psenen*^*+/+*^ fibroblasts (Fig. 3*E*). This contrasts with the higher activity in Aβ production from *Ncstn*^*−/−*^ fibroblasts, which was >3% of the wildtype production. This result confirms previous work that showed that in the absence of NCSTN some γ-secretase activity can still be measured (40). PSENEN on the other hand appears to be necessary to generate an active complex in cell culture. This is in line with an *in vitro* reconstitution experiment that demonstrated that PSENEN is needed to activate (wildtype) PSEN (34). AICD production was also completely abolished indicating that not only the γ-secretase cleavage of APP was disturbed but also the ε-cleavage (Fig. 3*D*).

Since PSENEN is considered to induce PSEN endoproteolysis, we wondered whether the lack of activity of the trimeric complex in *Psenen*^*−/−*^ fibroblasts was simply the consequence of the fact that PSEN fails to undergo endoproteolysis in the absence of PSENEN. To investigate this, we stably expressed the PSEN1 delta exon9 (PSEN1 ΔE9) mutant in the *Psenen*^*−/−*^fibroblasts. PSEN1 ΔE9 is active without the need for endoproteolytical processing (29, 43, 44). Even though PSEN1 ΔE9 was incorporated in the trimeric complex (Fig. 3, *A* and *B*), we failed to detect any γ-secretase activity (Fig. 3, *C* and *D*, lane indicated with *Psenen*^*−/−*^+ΔE9). In contrast, the PSEN1 ΔE9 mutant rescued γ-secretase activity in fibroblasts lacking PSEN1 and PSEN2, proving that it is indeed active in its noncleaved form (lane labeled Psen1&2^dKO^ + ΔE9.) This result indicates that PSENEN is not only essential for the endoproteolysis of PSEN but also plays a role in the active γ-secretase complex. Indeed a role of PSENEN in (i) the stabilization of the PSEN fragments after heterodimerization (25), (ii) the specificity of γ-secretase activity (38) and (iii) the accessibility or the affinity of γ-secretase to the substrate (45) has been reported.

Ahn *et al.* demonstrated that PSEN1 ΔE9 exhibits activity on its own, without the need for other γ-secretase components (34). However, in our experimental conditions, PSEN1 ΔE9 is present in the trimeric complex, which may change the properties of PSEN1 ΔE9. Therefore, we conclude that, in the context of the γ-secretase complex, PSENEN is needed for γ-secretase activity beyond endoproteolysis, considering that our assay conditions would have detected 1% of the wildtype activity.

### Scanning cysteine accessibility method analysis of PSENEN

We decided to use the scanning cysteine accessibility method, which has proven to be a valuable approach in the analysis of the structure–function of the PSEN1 subunit of the complex (22, 23, 24). We first generated a cysteine-free form of PSENEN (CL PSENEN) by replacing the unique cysteine (C15) in wildtype PSENEN by an alanine. The CL PSENEN variant was able to rescue γ-secretase complex formation in the *Psenen*^*−/−*^ fibroblasts as seen in blue native PAGE (Fig. 2, *C* and *D*). PSEN1 cleavage and NCSTN maturation were confirmed using SDS-PAGE/Western blotting (Fig. 2*D*). Importantly, as judged by APP CTF protein levels, γ-secretase proteolytic activity was restored to the same extent as in *Psenen*^*−/−*^ fibroblasts rescued with wildtype PSENEN (Fig. 2*D*). Therefore, we concluded that CL PSENEN is a suitable backbone for further analysis with the scanning cysteine accessibility method.

**Figure 4:**
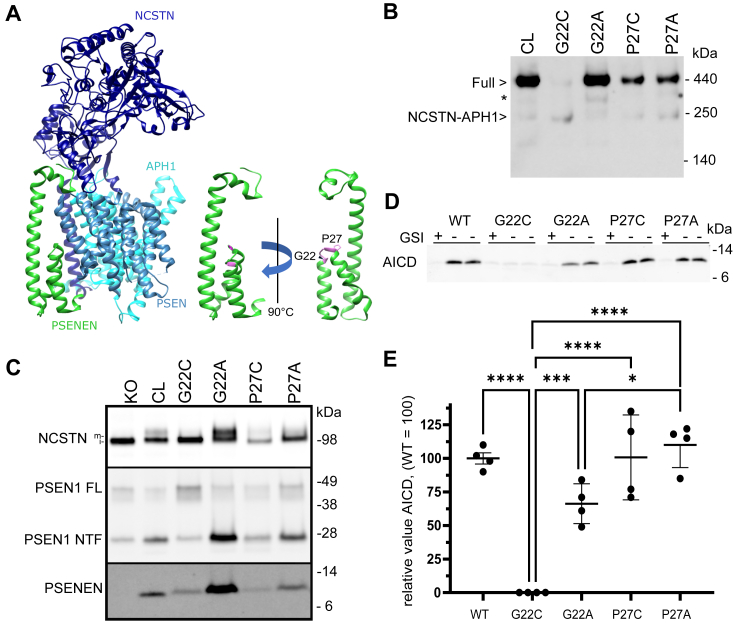
**The amino acids G22 and P27 of PSENEN are involved in complex formation and/or stabilization.***A*, structural representation of γ-secretase complex and positions of G22 and P27 (*pink*) in PSENEN (*green*). *B*, blue native PAGE/Western blot analysis of the different *Psenen* mutants. Expression of cysteine-less (CL) PSENEN in *Psenen*^*−/−*^ fibroblasts rescues full γ-secretase complex formation (lane 1). In contrast, expression of the G22C mutant compromises complex formation to a large extent (lane 2). Replacement of G22 with alanine (G22A) restores complex assembly (lane 3). Expression of the P27C mutant results in partial rescue of γ-secretase complex (lane 4). Replacement of P27 with an alanine (P27A) has similar effects as the P27C mutation (lane 5). ∗ indicates the detergent induced complex composed of PSEN1 CTF, APH1, and NCSTN (66). *C*, SDS-PAGE/Western blot analysis of *Psenen*^*−/−*^ fibroblasts expressing the G22 and P27 mutants. The PSENEN G22C mutant hardly rescues NCSTN maturation and decreases endoproteolysis of PSEN1 (lane 3), which agrees with the low levels of full complex seen in blue native PAGE analysis (*A*). Replacement of G22 by alanine (lane 4) restores NCSTN maturation and PSEN1 endoproteolysis to comparable levels as CL PSENEN (lane 2). Mutation of the P27 to cysteine or alanine results in very low PSENEN levels. Nevertheless, the levels of expression are sufficient to restore low levels of γ-secretase complex (lane 5,6). *D*, Western blot of AICD generated in microsomes isolated from *Psenen*^*−/−*^ fibroblasts transduced with the indicated PSENEN mutants in the presence or absence of 10 μM L-685,458 (GSI). Notice that all mutants apart from G22C rescue AICD generation. *E*, quantitation of four experiments as shown in D. AICD signal is equal to +GSI condition = background of the assay and AICD levels were normalized to PSEN1 NTF levels to obtain specific γ-secretase activity. Data of four experiments are presented in the graph. ∗∗∗∗: *p* < 0.0001; ∗∗∗*p* = 0.0008; ∗ *p* = 0.0235; ANOVA and Tukey multiple comparisons test.

**Figure 5:**
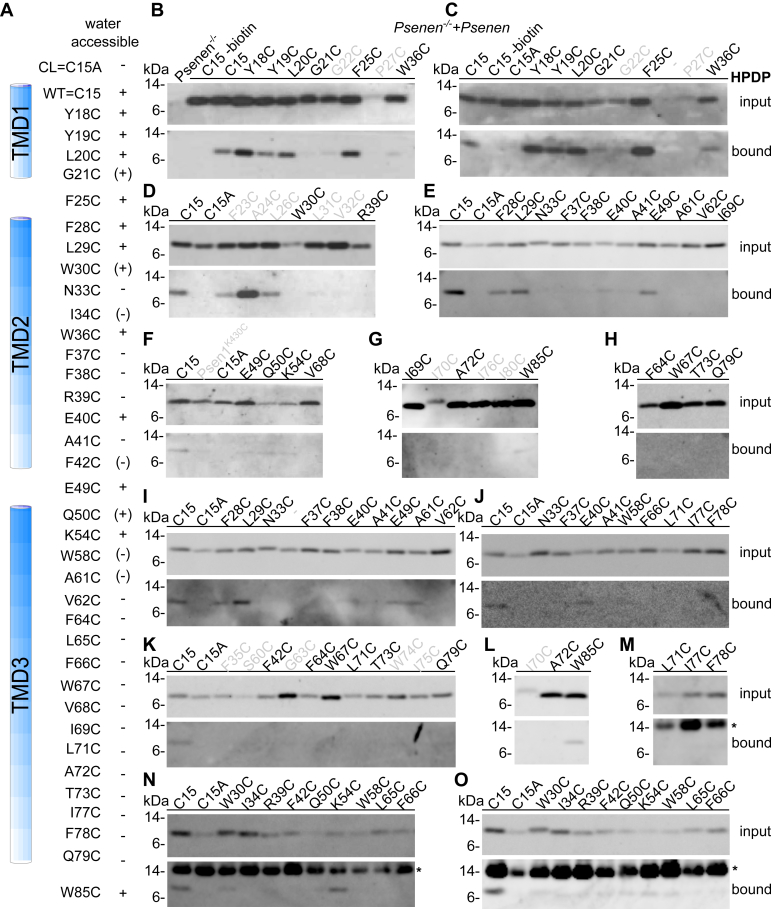
**Water accessibility of unique cysteines in PSENEN as demonstrated by HPDP.***PSENEN*^*−/−*^ fibroblasts were transduced with PSENEN containing the indicated single cysteine substitution. Intact cells were exposed to the membrane-permeable sulfhydryl-specific reagent EZ-linked Biotin-HPDP. *A*, schematic overview of the results. + and - indicate reactive and not reactive to the reagent, respectively. *B***–***O*, extracted biotinylated proteins were precipitated with neutravidin beads. The input and bound fractions were separated by 4 to 12% BisTris SDS-PAGE. The presence of PSENEN in the extract (input) and the bound fraction was detected using B126 antibody. Wildtype PSENEN (C15) with its luminal cysteine at the N terminus was used as a positive control, and cysteine-less PSENEN (C15A) was used as a negative control. Mutants further discussed in the current study are indicated in *black*. W30C is negative in *D* but positive in *N* and *O*. Mutants not interpreted in this study are labeled in *gray*, *gray* “-”indicates a lane with unlabeled sample, and ∗ indicates an unspecific band that originated from a new batch of neutravidin beads (only present in the bound fraction). Blots from different experiments are shown; amino acids in bold black were tested in at least two independent experiments. See also additional Fig. S1 for examples of the full blots used for *E* and *I* and for *N* and *O*.

**Figure 6:**
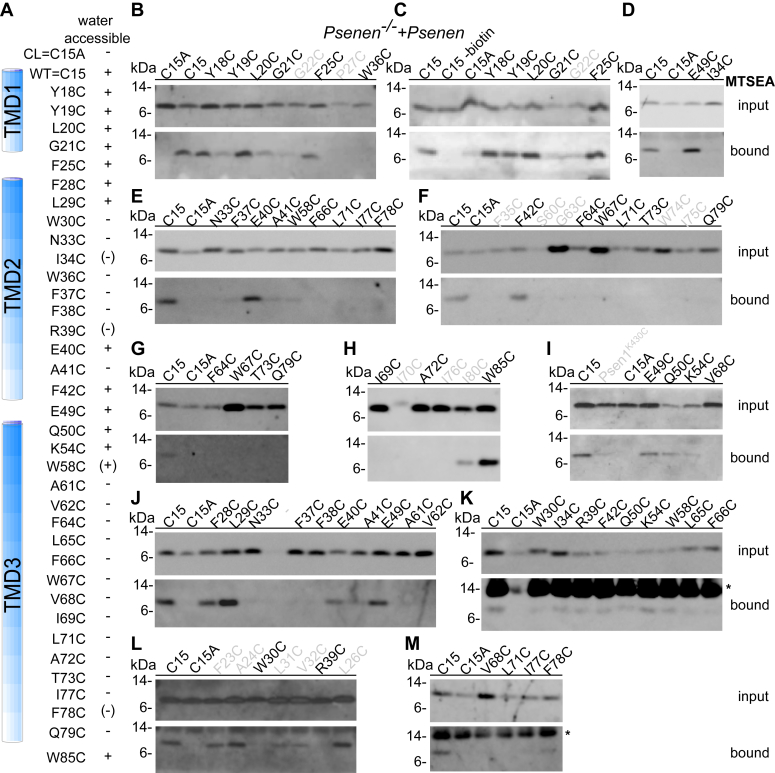
**Water accessibility of unique cysteines in PSENEN as demonstrated by MTSEA.***PSENEN*^*−/−*^ fibroblasts were transduced with PSENEN containing the indicated single cysteine substitution. Intact cells were exposed to the membrane-impermeable sulfhydryl-specific reagent MTSEA-biotin. *A*, schematic overview of the results. + and - indicate reactive and not reactive to the reagent, respectively. *B–M*, extracted biotinylated proteins were precipitated with neutravidin beads. The input and bound fractions were separated by 4 to 12% BisTris SDS-PAGE. The presence of PSENEN in the extract (input) and the bound fraction was detected using B126 antibody. Wildtype PSENEN (C15) with its luminal cysteine at the N terminus was used as a positive control, and cysteine-less PSENEN (C15A) was used as a negative control. Mutants further discussed in the current study are indicated in *black*. Mutants not interpreted in this study are labeled in gray. ∗ indicates an unspecific band that originated from a new batch of neutravidin beads (only present in the bound fraction).

We incorporated cysteine residues throughout PSENEN by site-directed mutagenesis (see Figure 4, Figure 5, Figure 6). The PSENEN mutants were stably expressed in the *Psenen*^*−/−*^ fibroblasts.

### Glycine 22 and Proline 27 are involved in complex formation and/or stabilization

Two cysteine mutants, *i.e.*, G22C and P27C, did not reconstitute fully γ-secretase levels (Fig. 4). Interestingly, although the G22C mutant was expressed to normal levels, complex formation, maturation, and activity were severely compromised (Fig. 4). The P27C mutant showed low expression levels (Fig. 4, *B* and *C*) but sufficient to partially restore NCTSN maturation and enzymatic activity (AICD generation in 4*D*). This suggests that the P27C mutant is unstable but once incorporated into the complex can replace wildtype PSENEN.

High-resolution cryo-EM data for the γ-secretase complex (PDB: 5A63) show that the N terminus of PSENEN is in the cytosol. Two helical domains (Asn8–Phe23 and Pro27–Leu43) are partially inserted in the membrane forming a U-turn structure and are followed by an intracellular loop (Val44–Gly49), a full membrane-spanning helical domain (Gln50–Tyr81), and a short extracellular C terminus (Arg82–Pro101). The PSENEN’s C–terminus is located at the interface between NCSTN and PSEN1 in this structure (Fig. 4*A*). Gly22 is located almost in the middle of the membrane at the end of the first partial-transmembrane domain, while Pro27 is present at the U-turn connecting the two partial transmembrane helices (Fig. 4*A*).

Glycine and proline are frequent amino acids in transmembrane domains (TMDs) of membrane proteins (46), and both contribute to transmembrane (TM) dynamics, but their specific roles depend on the local environment (22, 23). Glycine often functions as an interface between individual TM α-helices (47), and when positioned close to a Pro, it may enhance local dynamics and thus affect protein function (48). The structural data show that the conserved Gly22 and Pro27 residues contribute to the formation of the U-turn structure, and thus overall fold of PSENEN. Gly22 enables proximity between the two half-helical structures and the formation of a H-bonding with the backbone of Leu26. To investigate this further we introduced alanine at these two positions. In contrast to G22C, the G22A mutation displayed γ-secretase complex levels comparable with CL PSENEN (Fig. 4*C*). Furthermore, NCSTN maturation was rescued to a large extent (Fig. 4*C*) and cell-free activity assays demonstrated AICD production (Fig. 4, *D* and *E*). These results show that the backbone flexibility provided by G22 is not entirely essential for the function of the γ-secretase complex; however, the functional data also indicate that, while the short side chain of alanine (G22A) is tolerated, the larger cysteine side chain sterically disturbs helix–helix interactions in the PSENEN fold and thus affects the assembly and/or stability of the protease complex. The P27A mutation, in contrast, had the same effect on PSENEN levels and γ-secretase maturation and activity as the P27C mutation (Fig. 4*B*). The low levels of mutant PSENEN P27A that assemble into the enzyme complex display similar specific activity as CL PSENEN, indicating that the mutant assembles into functional γ-secretase complexes (Fig. 4, *D* and *E*). Prolines are usually found in irregular structures such as β-turns and α-helical capping motifs (49). In PSENEN, mutation of P27 to another amino acid results in loss of stability of PSENEN and the γ-secretase complex.

### Hydrophobic domain 1 and 2 are water accessible from the extracellular side

To delineate the solvent accessibility of the PSENEN amino acid residues, we tested the reactivity of the introduced cysteines to EZ-linked Biotin-HPDP (HPDP-biotin) (Figs. 5 and S1). HPDP-biotin is a membrane-permeable sulfhydryl-reactive reagent that can only react with free sulfhydryl groups exposed to a hydrophilic environment. The amino acids Cys15 (WT) and Y18C-G21C, F25C and F28C-W30C, W36C, all present in the partial TM hydrophobic domains 1and 2, were water accessible. In contrast, cysteines at positions N33, I34 (see also MTSEA experiment), F37, and R39 did not react with the probe. The results support a model in which part of the first two hairpin-forming hydrophobic domains of PSENEN (helix 1 and helix 2) are exposed to a hydrophilic environment. Furthermore, in agreement with the structural data, residues F42C, E49C, Q50C, K54C, and W58C connecting the hydrophobic domain 2 and TMD 3, as well as the extracellular W85C, were also labeled. In contrast, the A61C to Q79C residues in PSENEN were not labeled by the HPDP-biotin, indicating that this part of the full transmembrane domain (helix 3) is hydrophobic (Fig. 5).

**Figure 7:**
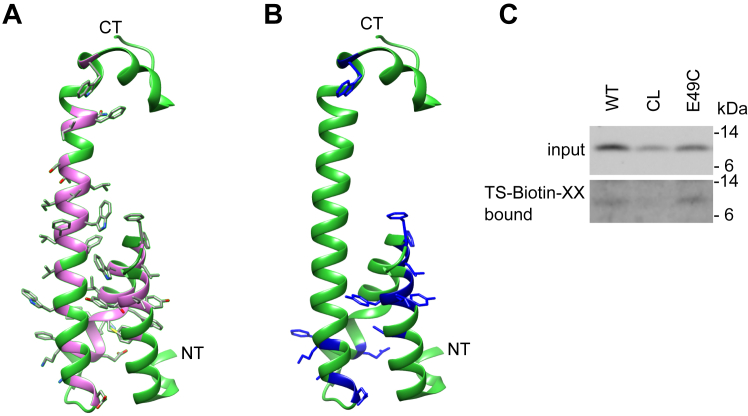
**Schematic representation of the scanning cysteine accessibility method results.***A*, three-dimensional model of PSENEN. The side chains of the amino acids targeted in the cysteine scan are shown. The purple areas indicate the positions of the amino acids that were targeted in the cysteine scan. *B*, three-dimensional model of PSENEN. The positions of amino acids in the backbone of the structure that are exposed to the hydrophilic environment as detected with MTSEA are indicated in *blue*. *C*, as a control for the reliability of the MTSEA reagent, intact cells were treated with the membrane-impermeable TS-Biotin-XX in the same way as in (*A*). The PSENEN cysteine mutant E49C shows reactivity to the impermeable TS-XX-Biotin, confirming the results of the MTSEA-biotin reagent.

Next, we evaluated the reactivity of cysteines to the membrane-impermeable MTSEA-biotin (Figs. 6 and 7, *A* and *B*). Remarkably, most residues in the partial TMD 1-2 of PSENEN that were labeled by the HPDP-biotin probe were also accessible by the MTSEA-biotin, with the exception of W30C and W36C. Residues F42C, E49C, Q50C, and K54C between HD2 and TM3 are labeled by the MTSEA-biotin reagent. The labeling pattern confirms that part of the HD1 and 2, together with the connecting loop and the beginning of TM3, is accessible from the extracellular/luminal site (Fig. 7*B*). The labeling of Cys49 was confirmed using another membrane-impermeable reagent (TS-XX-biotin) (Fig. 7*C*), which contains, in contrast to the neutral methanethiosulfonate group of MTSEA-biotin, a negatively charged thiosulfate group and is therefore even less likely to cross the cell membrane. Based on these findings, we speculate that the hydrophobic domains 1 and 2 and the linker with the transmembrane domain 3 are connected to the aqueous environment of the active site in PSEN. The transmembrane domain 3, in contrast, remains largely unlabeled (14 residues tested between A61 and Q79) by both biotin moieties, indicating that this part of PSENEN is embedded in a hydrophobic environment (Fig. 7*B*).

### Cross-linking studies locate E49 and PSEN1 CTF in close proximity

The catalytic site of γ-secretase is situated at the interface between PSEN NTF and PSEN CTF (21, 22, 23, 50) in a water-containing cavity. The accessibility results indicate that the N terminus containing the two hydrophobic domains of PSENEN relates to the catalytic pore in γ-secretase. To investigate this further, we performed bifunctional cross-linking assays involving a cysteine at E49, which is in the U-turn linker between the two hydrophobic domains.

**Figure 8:**
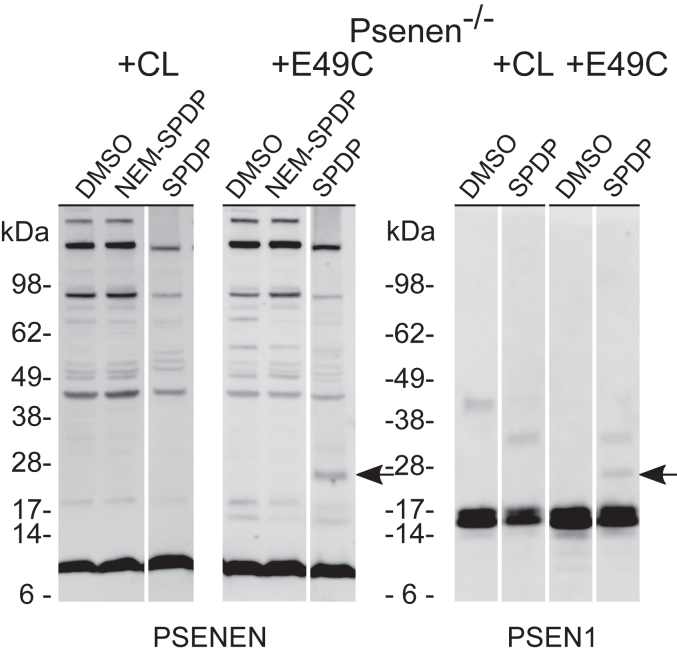
**Cross-linking studies reveal close proximity of E49 in PSENEN to PSEN1 CTF.** Membrane fractions of *Psenen^−/−^* fibroblasts transduced with cysteine-less (CL) PSENEN or PSENEN E49C were treated with the heterobifunctional cysteine-amine cross-linker SPDP (spacer arm, 6.8 Å). Blocking of the free sulfhydryl groups with NEM was performed as negative control. As additional negative control, cross-linker was omitted in the sample (DMSO). Protein extracts were separated in SDS-PAGE under nonreducing conditions on a 4 to 12% BisTris gel and visualized on Western blot with antibodies against γ-secretase components PSENEN or PSEN1. A band with a molecular weight of approximately 26 kDa was observed with PSENEN and PSEN1 CTF antibody, only in the samples transduced with the E49C mutant in the presence of cross-linker (*black arrow*). The samples were loaded on three different gels as indicated by the wide white space between the lanes. Lanes from the same gel are presented with a narrow white space. The original data used to assemble this figure are presented in Fig. S2.

Microsomal membrane fractions from the E49C PSENEN mutant were treated with SPDP, a heterobifunctional cross-linker with spacer arm of 6.8 Å, at 4 °C. SPDP conjugates primary amine (mainly lysines) and sulfhydryl (cysteines) groups of proteins. After cross-linking, conjugated products were separated by SDS-PAGE in nonreducing conditions and analyzed by Western blotting. Antibodies against PSENEN showed a higher mobility band for the PSENEN E49C mutant (Figs. 8, lane 6, arrow and S2). This band was also detected with an antibody against PSEN CTF (Figs. 8, lane 10, arrow and S2), while antibodies against other γ-secretase components (NCSTN, APH1, and PSEN1 NTF) did not react with this band. Moreover, the band was not observed when the free sulfhydryls were blocked with the alkylating agent N-ethylmaleimide (NEM) prior to the cross-linking reaction (Figs. 8 lane 5 and S2) or when reducing conditions were applied. No cross-linked products were detected with the cysteine-less (CL) PSENEN (Figs. 8 lanes 1–3, 7, 8 and S2). The molecular weight of the band (∼26 kDa) is in accordance with a cross-linked product between PSENEN and PSEN1 CTF. Therefore, our results show that the loop of PSENEN and the PSEN1 CTF are at a distance equal to or less than 6.8 Å from each other in the γ-secretase complex. Considering the available structural data, the cross-link may involve Cys49 in PSENEN and an amine group within the large intracellular (N-terminal) part of PSEN CTF. This flexible, unstructured region in PSEN CTF, not seen in the structure, is the only region that could approach the intracellular loop in PSENEN at the short distance reported by the cross-linking experiments. Alternatively, one would need to allude to large conformational changes in PSEN CTF to bring the loop in PSENEN to this short distance.

Until now, only PSENEN and PSEN modifications have been implicated in changes in γ-secretase activity (ratio changes) (15, 38). This implies that PSENEN may play a functional role in the γ-secretase activity, which is clearly corroborated by our experiments shown in Figure 3*E*. While a trimeric γ-secretase complex containing PSENEN but not NCSTN remains partially active (3–6% of the wildtype complex), the trimeric complex containing NCSTN but not PSENEN is completely inactive. Even when we expressed a functional mutant of PSEN1, PSEN1 ΔE9, which does not need endoproteolytical activation, the trimeric APH1–NCT–PSEN1 ΔE9 complex remained inactive, confirming that PSENEN is needed beyond the endoproteolysis that activates the enzyme.

Our previous report (18) has been retracted because of doubts raised by the editors regarding some of the figures. We provide here reassembled figures that should restore trust in the conclusions of our previous work. The Notch deficient phenotype of the *Psenen*^*−/−*^ mice (17, 51, 52, 53, 54, 55), the *Psenen*^*−/−*^ fibroblasts to study structure–function (56, 57, 58, 59), the role of PSENEN in complex formation (52, 54, 60, 61, 62), and the data of the cysteine scan (19, 28, 59) were used and cited in other publications, and based on the reported data here, these citations remain fully valid. One major change in the current article is the updated interpretation of the cysteine scan data presented in Figure 5, Figure 6, Figure 7 and S1. This interpretation takes fully into account the information regarding structure of the γ-secretase obtained by recent cryo-EM approaches, which show that PSENEN makes a reentrant loop and that the N and C termini of PSENEN face opposite sides of the cell membrane (19, 20). These data were not available when we published our original article but were used in the elegant work of Zhao *et al.* (28), which provided a correct interpretation of the structure. Our data reveal that the hydrophobic domains, helices 1 and 2, in PSENEN are inserted in the membrane but are surrounded by a polar environment. In contrast, the full membrane spanning TMD3 is mostly hydrophobic.

## Experimental procedures

### Generation of Psenen^−/−^ embryos

*Psenen*^*+/−*^ male and female mice were purchased from the Texas Institute for Genomic Medicine and coupled to obtain *Psenen*^*−/−*^ embryos. Mice were kept on a C57Bl6J background and housed in cages enriched with wood-wool and shavings as bedding and given access to water and food *ad libitum*. All experiments were approved by the Ethical Committee for Animal Experimentation at the University of Leuven (KU Leuven).

### Whole mount *in situ* hybridization

Embryos were isolated from the mother at the indicated embryonic days and fixed in 4% paraformaldehyde in PBS. After dehydration to 100% methanol and bleaching in 6% hydrogen peroxide for 1 h at room temperature, embryos were rehydrated in 100% PBS + 0.1% Tween. The embryos were treated with 10 μg/ml proteinase K for 5 min and post-fixated in 0.2% gluteraldehyde and 4% paraformaldehyde. Prehybridization was done in 50% formamide and 5x saline–sodium citrate pH 4.5 complemented with 50 μg/ml yeast RNA and 50 μg/ml porcine heparin during 1 h at 60 °C, after which the prehybridization mix was replaced for the hybridization mix containing 1 ng/μl digitonin-labeled riboprobes against Notch signaling pathway components as indicated. After overnight incubation in the hybridization mix at 60 °C, the embryos were washed extensively in PBS with 0.1% Tween and treated with RNase A (100 mg/ml) for 15 min.

Blocking was performed in 2% Boehringer Blocking Reagent and 20% fetal bovine serum in MABT buffer (100 mM maleic acid, 150 mM NaCl, pH 7.5, 0.1% Tween). After blocking, the embryos were treated with an alkaline phosphatase–coupled anti-digitonin antibody (final concentration 1/2000) overnight at 4 °C. On the next day, the embryos were washed in MABT and treated with levamisole (2 mM). BM purple, a chromogenic substrate for alkaline phosphatase, was added, and the staining was visualized by light microscopy.

### Generation of Psenen^−/−^ fibroblasts and cell culture

Fibroblasts with the different genotypes as indicated were generated from E9.5 embryos and immortalized by transfection with the plasmid pMSSVLT, driving expression of the large T-antigen. Immortalized mouse embryonic fibroblasts were cultured in Dulbecco's modified Eagle's medium/F-12 containing 10% fetal bovine serum (Sigma).

### Generation of PSENEN mutants and corresponding stable cell lines

All mutations in PSENEN were generated with the XL site-directed mutagenesis kit (Stratagene) and confirmed by DNA sequence analysis. Retroviruses were generated by cotransfecting pMSCVpuro vector containing PSENEN and the PIK helper plasmid into HEK293 cells. Viral particles harvested at 48 h post transfection were used to infect *Psenen*^*−/−*^ fibroblasts at 30 to 40% confluency. Transduced cells were selected with 5 μg/ml puromycin.

### Antibodies

Polyclonal antibodies against mouse PSENEN (B126.2), PSEN1 NTF (B19.3), APH1A (B80.2), and APP C terminus (B63.3) and monoclonal 9C3 against the C terminus of NCTSN have been described (63, 64). The following antibodies were purchased: anti-N-cadherin (BD Biosciences), anti-NICD (cleaved Notch1 Val-1744) (Cell Signaling Technologies), mAb 9E10 (Sanver Tech), and MAB5232 against PSEN1 CTF (Chemicon). The anti-Flag M2 antibody was purchased from Sigma-Aldrich.

### SDS-PAGE

Total cell lysates were prepared in lysis buffer (5 mM Tris-HCl pH 7.4, 250 mM sucrose, 1 mM EGTA, 1% Triton X-100, and complete protease inhibitors [Roche Applied Science]). Post-nuclear fractions were taken and protein concentrations were determined using standard bicinchoninic acid assay (Pierce). Protein, 20 μg, was separated on 4 to 12% BisTris gels and transferred to nitrocellulose membranes to perform Western blot analysis. Signals were detected using chemiluminescence with Renaissance (PerkinElmer) and developed either by X-ray film or LAS-3000 (Fuji). Quantification of the Western blots was done with Fiji (ImageJ).

### Blue native PAGE

Microsomal membrane fractions were prepared in lysis buffer containing 0.5% dodecylmaltoside, 20% glycerol, and 25% BisTris/HCl pH7. Upon ultracentrifugation (55,000 rpm), supernatant was taken, protein concentrations were measured, and 5 μg of protein was supplemented with sample buffer. Samples were loaded on a 5 to 16% polyacrylamide gel and run for 4 h at 4 °C. Subsequently, the gel was incubated with 0.1% SDS for 10 min at room temperature and transferred to a polyvinylidene difluoride membrane. Membranes were destained in water/methanol/acetic acid (60/30/10, v/v/v) and incubated with antibodies to detect γ-secretase complex.

### Water accessibility assay

Cells were plated in 10-cm dishes, washed with PBS, and treated with the biotinylated sulfhydryl-specific reagents for 30 min at 4 °C. In case of pretreatment with Sodium (2-Sulfonatoethyl) methanethiosulfonate (MTSES), cells were incubated with MTSES or dimethyl sulfoxide (DMSO) for 30 min at 4 °C before treatment with the biotinylated reagent. After extensive washing in PBS to remove unbound reagent, cells were collected and lysed in 25 mM Hepes pH 8, 150 mM NaCl, 2 mM EDTA, 1% Triton X-100. Biotinylated proteins in the total cell lysates were precipitated with immobilized NeutrAvidin protein beads (Pierce). Bound proteins were eluted by boiling in Nu-Page sample buffer, and SDS-PAGE was performed as mentioned above. PSENEN was detected in input and bound fractions.

Sulfhydryl-specific reagents used: EZ-Link Biotin-HPDP (N-(6-(Biotinamido)hexyl)-3′-(2′-pyridyldithio)-propionamide, Pierce) (200 μM), MTSEA-biotin (N-biotinylaminoethyl-methanethiosulfonate, Biotium) (500 μM), TS-XX-biotin ethylenediamine (biotin-XX ethylenediamine thiosulfate, sodium salt, Biotium) (500 μM), and MTSES (Sodium (2-Sulfonatoethyl)methanethiosulfonate, Biotium) (200 μM).

### Cross-linking

Cross-linking studies were performed by incubating microsomal membrane fractions with the heterobifunctional amine-sulfhydryl-reactive cross-linker SPDP (*N*-Succinimidyl-3-(2-pyridyldithio)-propionate) (1 mM). As negative controls, membranes preincubated with 10 mM N-ethylmaleimide (NEM)/10 mM EDTA or membranes incubated with DMSO were used.

Reactions were quenched with NEM/EDTA. After centrifugation at 55,000 rpm, membrane pellets were solubilized in 5 mM Tris-HCl pH 7.4, 250 mM sucrose, 1 mM EGTA, 1% Triton X-100; equal amounts of proteins were separated in SDS-PAGE in nonreducing conditions and detected by Western blotting using antibodies against γ-secretase components.

### Cell-free APP processing assay

Cell-free assays were performed as described by Kakuda *et al.* with some minor modifications (65). Briefly, microsomal membrane fractions solubilized in 1% CHAPSO were mixed with recombinant APPC99-3xFlag substrate (0.5 μM final concentration), 0.0125% phosphatidylethanolamine, 0.1% phosphatidylcholine, and 2.5% DMSO. Reactions were incubated at 37 °C for 3 h. AICD was detected by Western blot analysis with the anti-Flag M2 antibody and Aβ species by the AlphaLISA technique (see below).

Aβ and AICD levels were normalized to the amounts of γ-secretase complex in the *in vitro* assay, which were estimated from the PSEN1 NTF levels.

### Cell-based APP processing assay

Fibroblasts were infected with an adenoviral vector (Ad5) bearing human APP-695 containing the Swedish mutation. The cells were then cultured in Dulbecco's modified Eagle's medium supplemented with 0.2% fetal bovine serum for 16 h, and the conditioned medium was collected and used to analyze APP processing. Aβ_40_ and Aβ_42_ levels were quantified by AlphaLISA (see below) and sAPP levels by SDS-PAGE and Western blot analysis. Cell lysates were prepared, and APP full length and APP CTF were analyzed by SDS-PAGE followed by Western blot analysis. Aβ levels were normalized to infection efficiency, quantified from the sAPP expression levels. APP CTFs were normalized to APP full length.

### Cell-based Notch processing assay

Fibroblasts were infected with an adenoviral vector (Ad5) containing Myc-tagged NotchΔE. At 24 h post infection, 10 μM lactacystin was added to the cultures and after 4 h cell lysates were prepared. NICD and NotchΔE levels were estimated by Western blot analysis using a neoepitope (cleaved Notch1 Val-1744) and an anti-myc antibody, respectively.

NICD levels were normalized to the levels of infection efficiency (levels of NotchΔE).

### Mycoplasma

All cells lines were screened for absence of mycoplasma contamination (MycoAlert, Westburg).

### AlphaLISA (PerkinElmer)

Conditioned medium was mixed with PBS supplemented with 0.1% casein, streptavidin-coated AlphaLISA donor beads, and biotinylated antibody against the neoepitope of Aβ_40_ or Aβ_42_. After overnight incubation at 4 °C, acceptor-beads coupled to an antibody against the N terminus of Aβ were added. After 1 h incubation, light emission (615 nm) was detected upon laser excitation at 680 nm.

### Statistical analysis

Data from four experiments were used for calculation of *p*-values using one-way ANOVA and Tukey multiple comparisons test.

## Data availability

The cell lines and raw data described in this paper are available upon request.

## Supporting information

This article contains supporting information.

## Conflict of interest

B. D. S. is consultant for several pharmaceutical companies and is scientific founder of Augustine TX and founder and (minor) shareholder of Muna TX.

## Supporting information

Functional and topological analysis of PSENEN_Sup
