# Supplementary material for: Functional and topological analysis of PSENEN, the fourth subunit of the γ-secretase complex
Source: J Biol Chem. 2023 Dec 10;300(1):105533. doi: 10.1016/j.jbc.2023.105533 (PMC10790097; doi:10.1016/j.jbc.2023.105533)
Supplement: Functional and topological analysis of PSENEN_Sup [file mmc1.pdf]

Functional and topological analysis of PSENEN, the fourth subunit of the γ-secretase complex

Lutgarde Serneels[1,2,1], Leen Bammens[1,2*], An Zwijsen[3], Alexandra Tolia[1,2], Lucía Chávez-Gutiérrez[1,2] and Bart De Strooper[1,2]

[1] Laboratory for the Research of Neurodegenerative Diseases, VIB Center for Brain & Disease Research, VIB, Leuven, Belgium
[2] Department of Neurosciences and Leuven Brain Institute, KU Leuven, Leuven, Belgium
[3]*Laboratory of Developmental Signaling, Center for Human Genetics, KULeuven, Belgium*

Address correspondence to: Bart De Strooper, Herestraat 49 3000 Leuven, Belgium
T: +3216346227; F: +3216347181; E-mail: Bart.DeStrooper@kuleuven.be

Supporting information

Fig. S1

Psenen$^{-/-}$ fibroblasts expressing the indicated PSENEN cysteine mutants were treated with membrane permeable sulfhydryl-specific reagent EZ-linked Biotin-HPDP. Cell lysates from $5x10^6$ cells were solubilized in 1000µl lysis buffer, 20 µg of total protein was used for analysis of reconstitution of γ-secretase complex and to control for PSENEN expression using 4-12% Bis-Tris SDS page run in MES buffer (input). Five hundred µg of the lysate was incubated with neutravidin beads and bound fractions were boiled in SDS and also analyzed on 4-12% Bis-Tris SDS page. As indicated in the figures, blots were cut in three parts (indicated with scissor). The upper part (I) was stained with 9C3 against NCSTN, the middle part (II) was stained with B19 against PSEN1 and the third part (III) was stained with PSENEN B126 antibody. After washes, membranes were incubated with secondary antibodies and blots were reassembled as shown. ECL-signals were detected either by X-ray film or using the LAS3000 detection system. We show here the four experiments (experiment 1 to 4) used to assemble panels E and I and N and O from Fig. 5.

Each experiment is presented in the same way. The two upper panels show the blots as they were developed with the cutting line to generate the pieces of the blot (I-III) indicated. At the right side the different subunits stained with the antibodies mentioned above are indicated.

The middle panels are the same as the upper panels, but the part of the blot used for the figure in the main manuscript is indicated.

The lower panel is the assembled one shown in the manuscript. The upper panels E and I and upper panel N and O, respectively are showing the input materials as control. The lower panel shows the experimental part i.e. the signal of the cross-linked biotin form different experiments.

[1] The two first authors contributed equally and share the first authorship.

Experiment 1 leading to panel E

5E

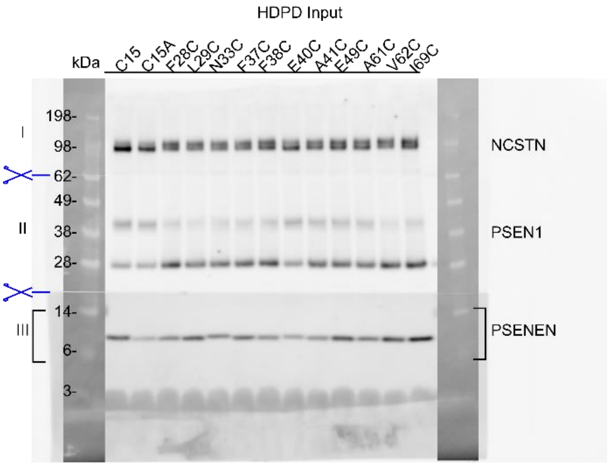

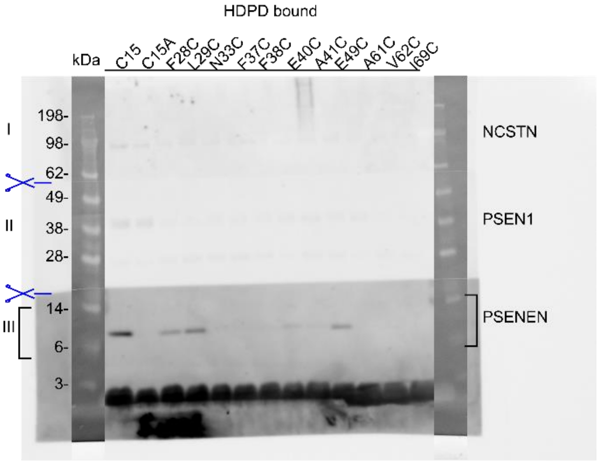

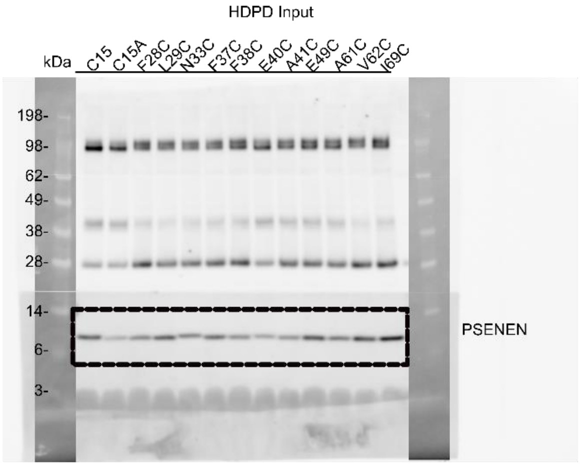

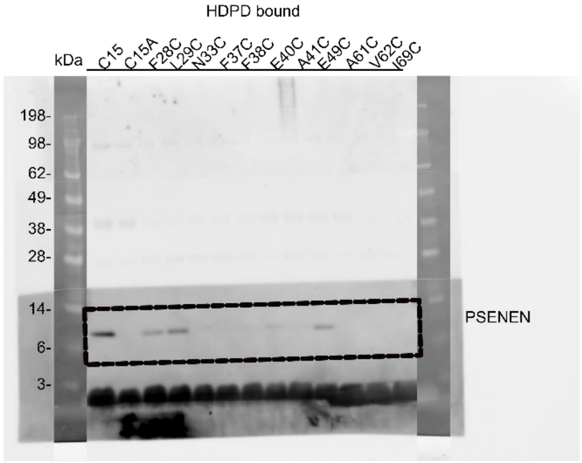

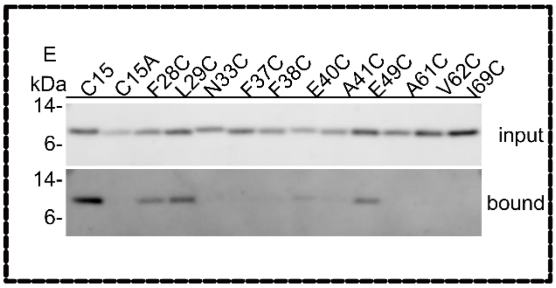

Experiment 2 leading to panel I

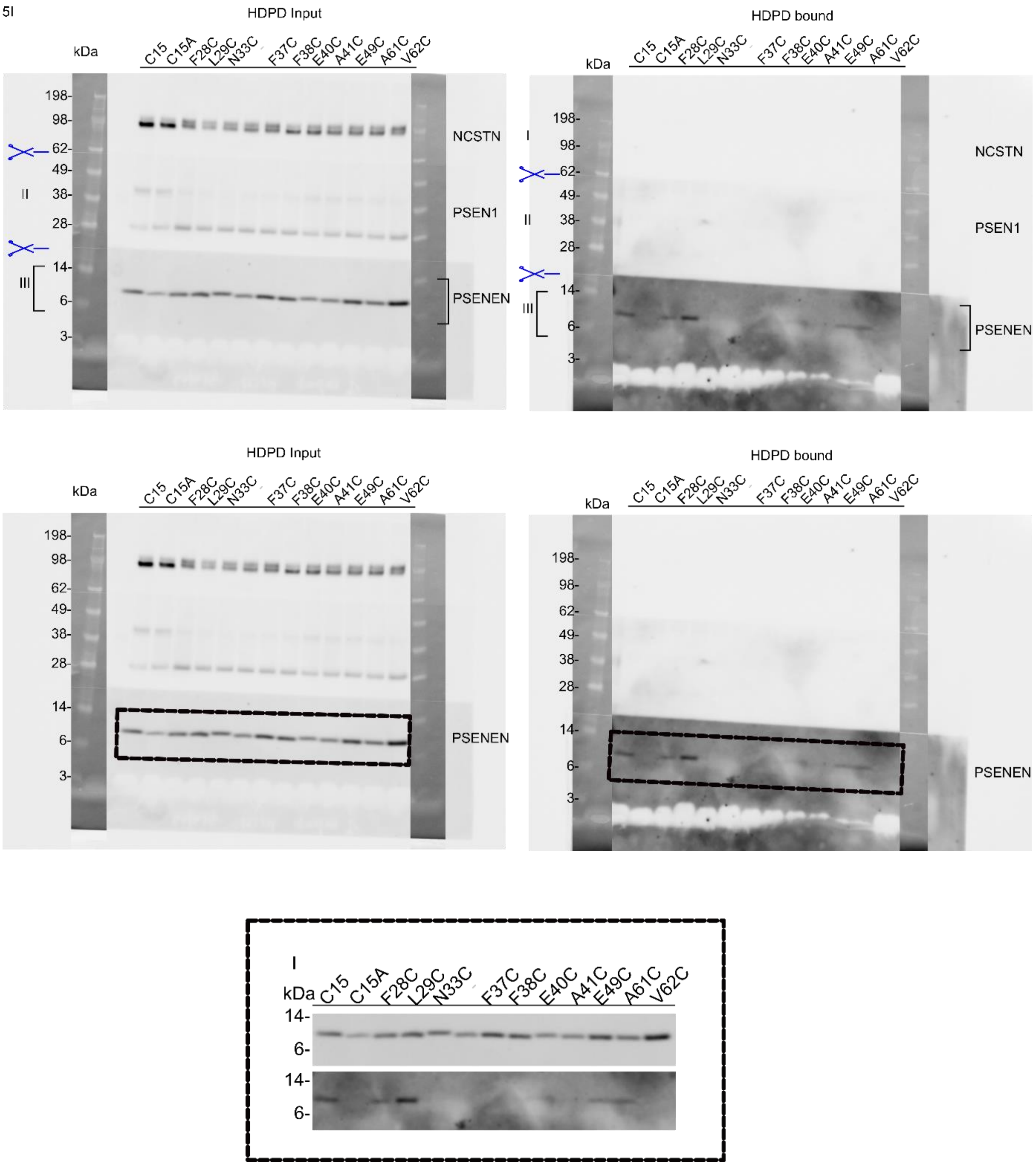

Experiment 3 leading to panel N

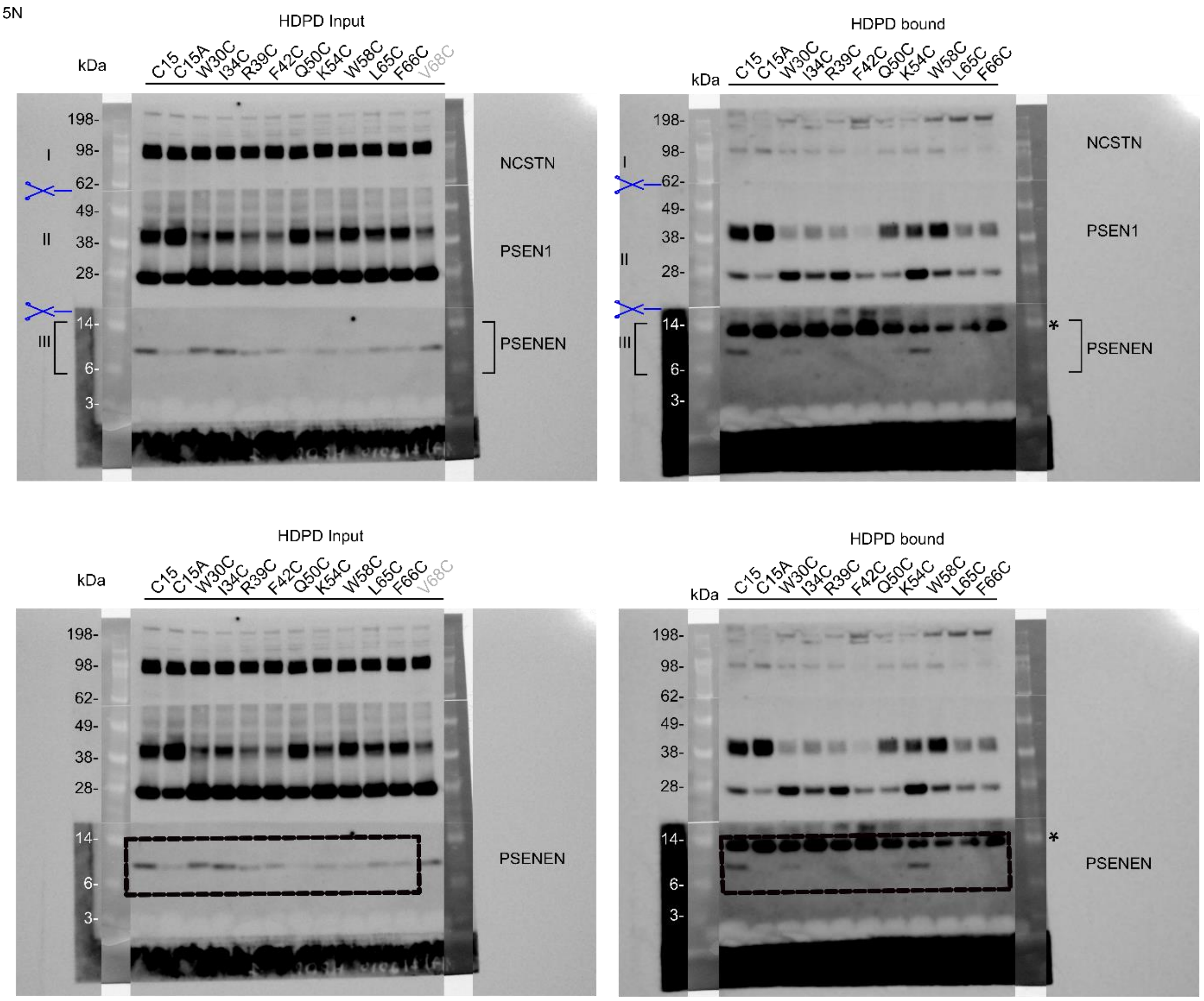

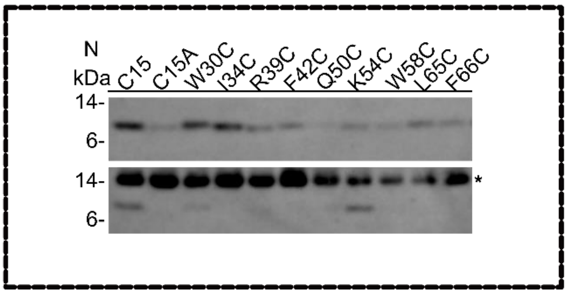

Experiment 4 leading to panel 0

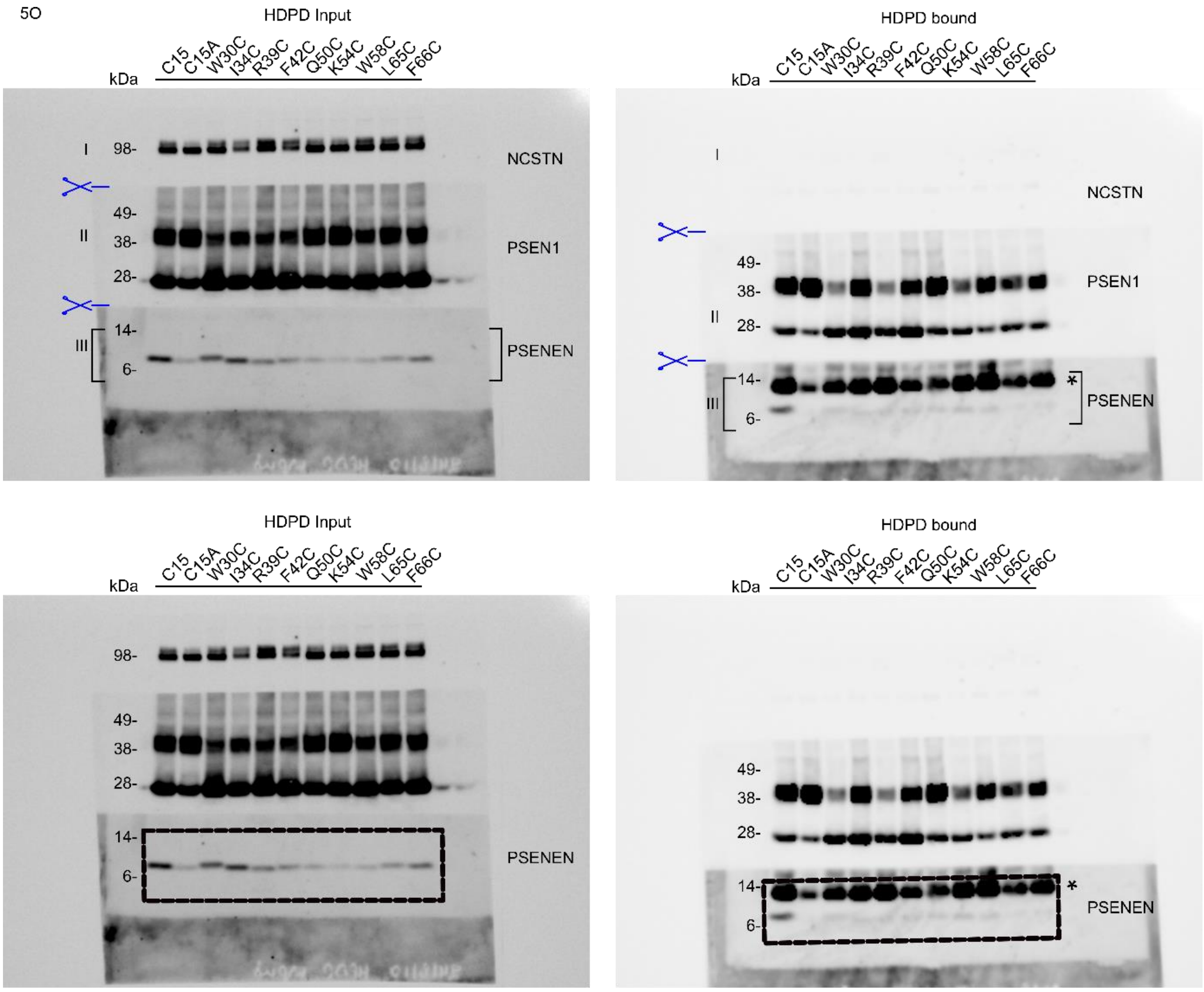

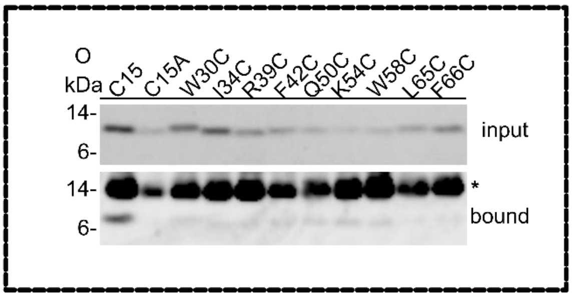

Fig. S2

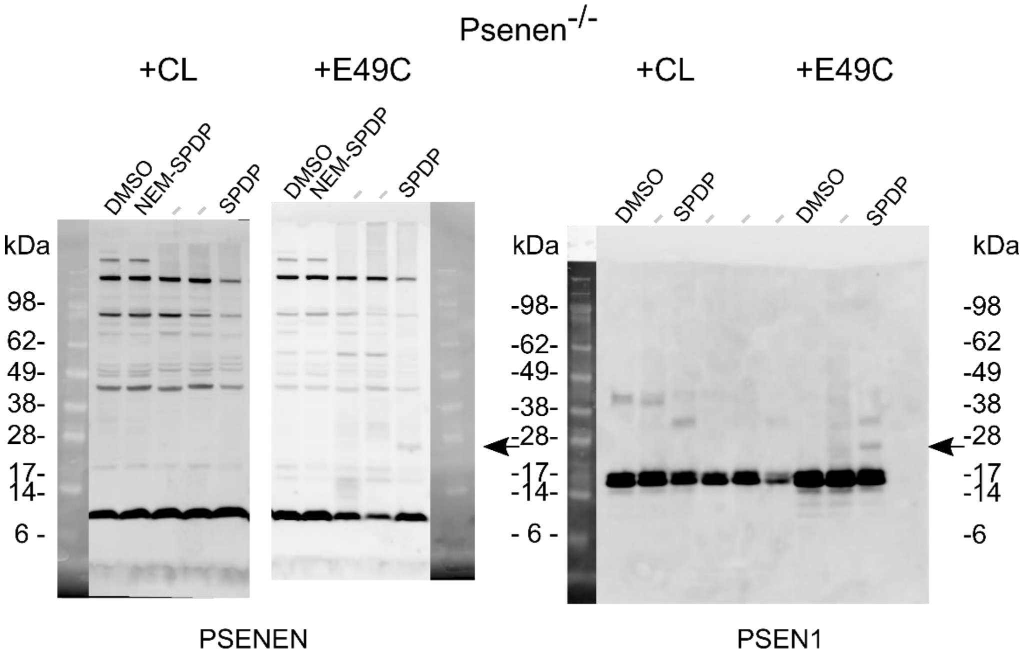

Fig. S2 Raw data used to compile Fig. 8.

The samples presented in Fig. 8 were loaded on three gels. The molecular weight markers, the labels of the lanes and western blot results are shown. The lanes indicated with “-“ are not relevant for the study.
